# Supplementary material for: A heat-shock inducible system for flexible gene expression in cereals
Source: Plant Methods. 2020 Oct 14;16:137. doi: 10.1186/s13007-020-00677-3 (PMC7557097; doi:10.1186/s13007-020-00677-3)
Supplement: Supplementary file 1 — Additional file 1. Contains gene sequences of NAM-B1 and Cre which were domesticated for use in cloning. [file 13007_2020_677_MOESM1_ESM.docx]

Additional File 1: Gene Sequences

Domesticated *NAM-B1* sequence

The TtNAM-B1 gene sequence was domesticated to remove any *Bbs*I or *Bsa*I sites. One *Bbs*I site was identified (highlighted in yellow in the third exon), and was domesticated from “GTCTTC” to “ATCGTC”, removing the enzyme cut site but retaining the correct amino acid sequence. Exons are coloured, with the splice sites highlighted in red. The exonic sequence is based on the sequence of *NAM-B1* from *T. turgidum* ssp. *dicoccoides*, while the intronic sequence is taken from the non-functional copy of *NAM-B1* present in Chinese Spring.

>full-length domesticated *NAM-B1*

**ATG**GGCAGCTCCGACTCATCTTCCGGCTCGGCGCAAAAAGCAACGCGGTATCACCATCAGCATCAGCCGCCGCCTCCGCAGCGGGGCTCGGCGCCGGAGCTCCCGCCGGGCTTCCGGTTCCACCCGACGGACGAGGAGCTGGTGGTGCACTACCTCAAGAAGAAGGCCGACAAGGCGCCGCTCCCCGTCAACATCATCGCCGAGGTGGATCTCTACAAGTTCGACCCATGGGAGCTCCCCGGTATGTTATGTCTATCTCGTCGGCCGGCCGTGCTTACTTTATCAAGCGCCGCAAATTTTAGGTGCAATTAAATAATCGAATAATCCATCCATCTCATGCTTATACTCCTGTGCACAAGTAGTATTTTTATATTCTTCCAGTACACATGTGTGTAGATGGTTTATGTATGTGATCCTGTCGTGCTTGTTCATGCGCTCGGGATCCGGATCCATCAGAGAAGGCGACCATCGGGGAGCAGGAGTGGTACTTCTTCAGCCCGCGCGACCGCAAGTACCCCAACGGCGCGCGGCCGAACCGGGCGGCGACGTCGGGGTACTGGAAGGCCACCGGCACGGACAAGCCTATCCTGGCCTCGGGGACGGGGTGCGGCCTGGTCCGGGAGAAGCTCGGCGTCAAGAAGGCGCTCGTGTTCTACCGCGGGAAGCCGCCCAAGGGCCTCAAAACCAACTGGATCATGCACGAGTACCGCCTCACCGACGCATCTGGCTCCACCACCGCCACCAACCGACCGCCGCCGGTGACCGGCGGGAGCAGGGCTGCTGCCTCTCTCAGGGTACGTACACGTGTCGATCGCACGGTCTAGCAGTATTTAATTGCTCTCCAGCTTAATTAGGGTATTGTTGATGGTTGATGAAGTTGGACGACTGGGTGCTGTGCCGCATCTACAAGAAGATCAACAAGGCCGCGGCCGGCGATCAGCAGAGGAACACGGAGTGCGAGGACTCCGTGGAGGACGCGGTCACCGCGTACCCGCTCTATGCCACGGCGGGCATGACCGGTGCAGGTGCGCATGGCAGCAACTACGCTTCACCTTCACTGCTCCATCATCAGGACAGCCATTTCCTGGACGGCCTGTTCACAGCAGACGACGCCGGCCTCTCGGCGGGCGCCACCTCGCTGAGCCACCTAGCAGCGGCGGCGAGGGCAAGCCCGGCTCCGACCAAACAGTTTCTCGCCCCGTCATCGTCGACCCCGTTCAACTGGCTCGATGCGTCACCAGTCGGCATCCTCCCACAGGCAAGGAATTTTCCTGGGTTTAACAGGAGCAGAAACGTCGGCAATATGTCGCTGTCATCGACGGCCGACATGGCTGGCGCAGTGGACAACGGTGGAGGCAATGCGGTGAACGCCATGTCTACCTATCTTCCCGTGCAAGACGGGACGTACCATCAGCAGCATGTCATCCTCGGCGCTCCGCTGGTGCCAGAAGCCGCCGCCGCCACCTCTGGATTCCAGCATCCCGTTCAAATATCCGGCGTGAACTGGAATCCC**TGA**

Domesticated Cre-U5-Cre recombinase sequence

The Cre recombinase sequence was domesticated to remove any *Bbs*I or *Bsa*I sites. One BbsI restriction site was identified, and both domesticated from “CTTCTG” to “CTCCTG” via a silent T/C mutation. The U5 intron sequence (purple) was obtained from pICSL80006 (TSL SynBio), with exons coloured and splice sites highlighted in red.

>NC_005856.1:436-1467 Enterobacteria phage P1, complete genome

atgtccaatttactgaccgtacaccaaaatttgcctgcattaccggtcgatgcaacgagtgatgaggttcgcaagaacctgatggacatgttcagggatcgccaggcgttttctgagcatacctggaaaatgctcctgtccgtttgccggtcgtgggcggcatggtgcaagttgaataaccggaaatggtttcccgcagaacctgaagatgttcgcgattatcttctatatcttcaggtaagtttctgcttctacctttgatatatatataataattatcattaattagtagtaatataatatttcaaatatttttttcaaaataaaagaatgtggtatatagcaattgcttttctgtagtttataagtgtgtatattttaatttataacttttctaatatatgaccaaaatttgttgatgtgcaggcgcgcggtctggcagtaaaaactatccagcaacatttgggccagctaaacatgcttcatcgtcggtccgggctgccacgaccaagtgacagcaatgctgtttcactggttatgcggcggatccgaaaagaaaacgttgatgccggtgaacgtgcaaaacaggctctagcgttcgaacgcactgatttcgaccaggttcgttcactcatggaaaatagcgatcgctgccaggatatacgtaatctggcatttctggggattgcttataacaccctgttacgtatagccgaaattgccaggatcagggttaaagatatctcacgtactgacggtgggagaatgttaatccatattggcagaacgaaaacgctggttagcaccgcaggtgtagagaaggcacttagcctgggggtaactaaactggtcgagcgatggatttccgtttctggtgtagctgatgatccgaataactacctgttttgccgggtcagaaaaaatggtgttgccgcgccatctgccaccagccagctatcaactcgcgccctggaagggatttttgaagcaactcatcgattgatttacggcgctaaggatgactctggtcagagatacctggcctggtctggacacagtgcccgtgtcggagccgcgcgagatatggcccgcgctggagtttcaataccggagatcatgcaagctggtggctggaccaatgtaaatattgtcatgaactatatccgtaacctggatagtgaaacaggggcaatggtgcgcctgctggaagatggcgattaggcttatatgaagatgaagatgaaatatttggtgtgtcaaataaaaagcttgtgtgcttaagtttgtgtttttttcttggcttgttgtgttatgaatttgtggctttttctaatattaaatgaatgtaagatctcattataatgaataaacaaatgtttctataatccattgtgaatgttttgttggatctcttctgcagcatataactactgtatgtgctatggtatggactatggaatatgattaaagataag

ER-targeting sequences

HDEL sequence; located at the C-terminus of the eGFP protein:

catgatgagctt

ER targeting sequence; located at the N-terminus of the eGFP protein:

aaaactaatctttttctctttctcatcttttcacttctcctatcattatcctcggccgaattc
