# Supplementary material for: A heat-shock inducible system for flexible gene expression in cereals
Source: Plant Methods. 2020 Oct 14;16:137. doi: 10.1186/s13007-020-00677-3 (PMC7557097; doi:10.1186/s13007-020-00677-3)
Supplement: Supplementary file 2 — Additional file 2. Contains all Additional Figures and Tables referred to in the text. [file 13007_2020_677_MOESM2_ESM.docx]

**Additional Figures and Tables**


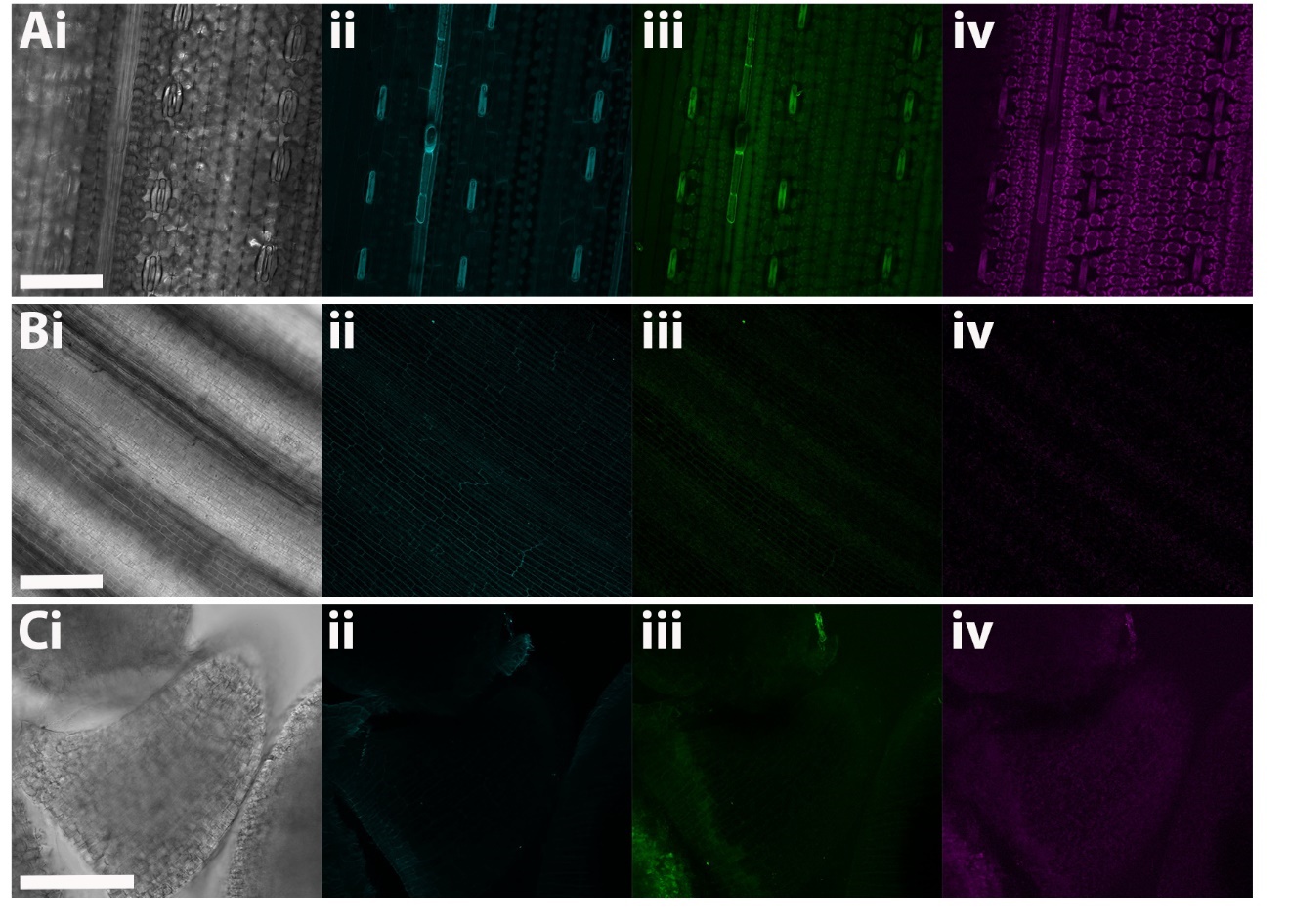


**Figure S1: Screening of wild-type barley tissue for endogenous fluorescence.** *A-C.i:* bright field channels. *ii:* CyPET channel. *iii:* eGFP channel. *iv:* mCHERRY channel. *A.i-iv*: images of mature barley leaf tissue (blade); *B i-iv*: images of young barley leaf tissue; *C i-iv:* images of a young wild-type barley lemma. Gain > 120 for each tissue. Scale bars are 100µm.

**
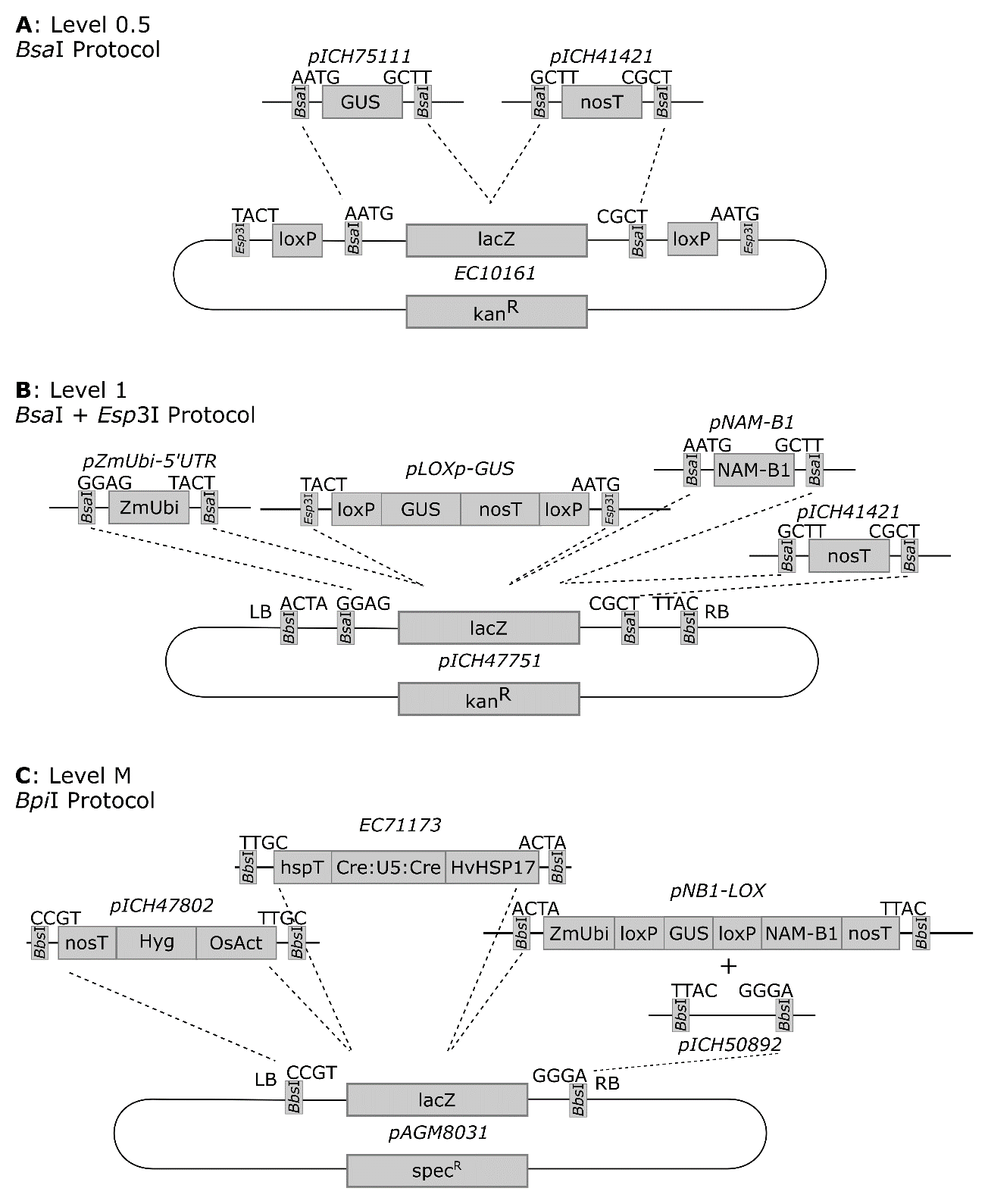
**

**Figure S2: Construction of the wheat construct.** A) The reporter gene GUS (pICH75111) and the *nos* terminator (pICH41421) were cloned into the Level 0 vector EC10161, using the Type IIS restriction enzyme *Bsa*I. The resulting Level 0.5 vector contains loxP sites flanking the inserted reporter gene and terminator. B) The maize Ubiquitin promoter *ZmUbi* with a 5’ UTR sequence (from pICSL12009, cloned into pUAP1), *lox*P-flanked GUS (pLOXp-GUS), domesticated *NAM-*B1 in the universal acceptor pUAP1 (pNAM-B1), and the *nos* terminator (pICH41421) are cloned into a Level 1, position 3 vector (pAGM8031) in a reaction involving both *Bsa*I, for the canonical Level 0 and Level 1 parts, and *Esp*3I, for the *lox*P-containing Level 0.5 vector (see A).C) The hygromycin selection cassette (pICH47802), the HvHSP17::Cre-U5-Cre construct (EC71173), and the pNB1-LOX construct are cloned into a Level M, position 1 construct (pAGM8031), along with the position 4 end linker (pICH50892), using the Type IIS restriction enzyme *Bpi*I.


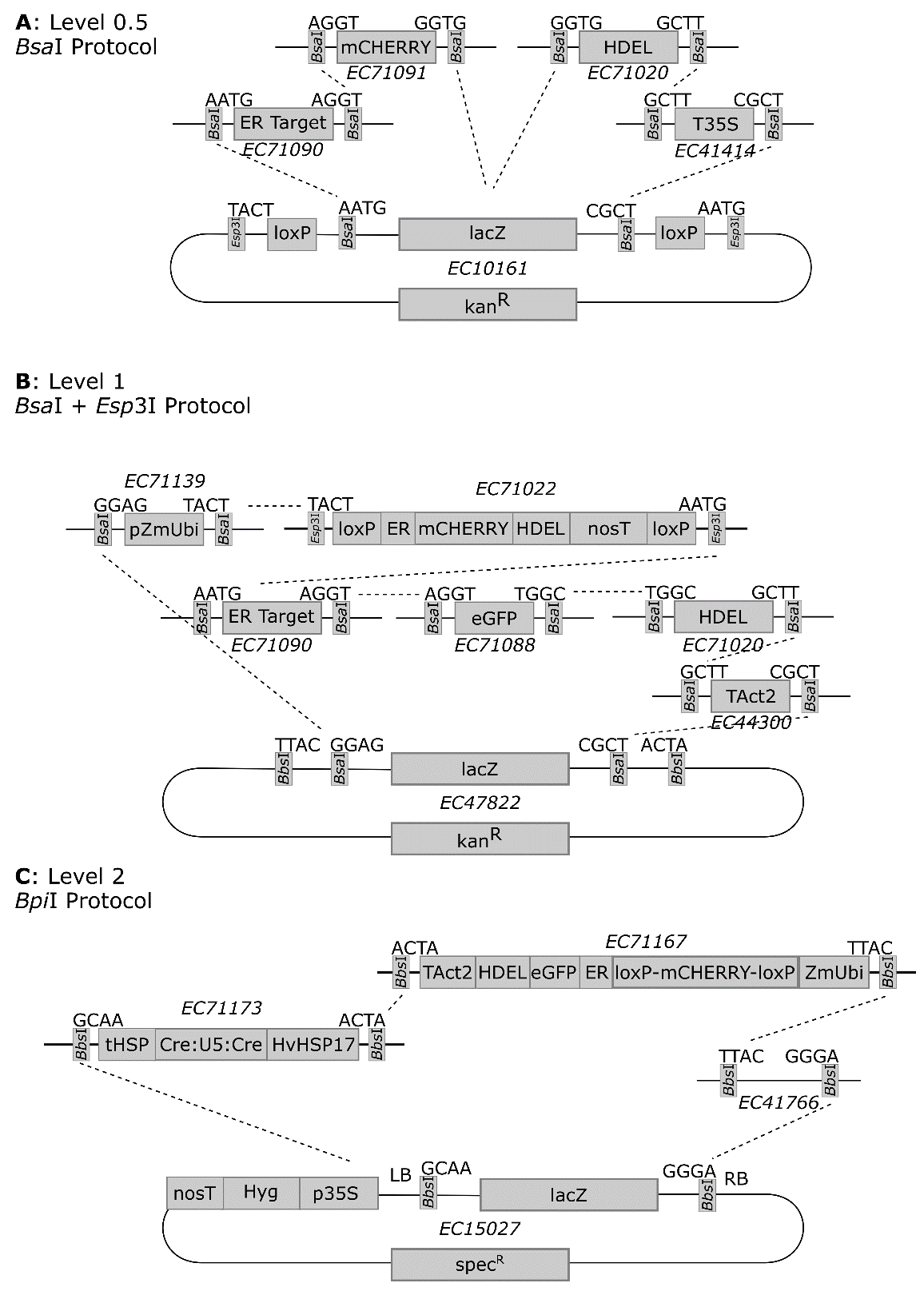


**Figure S3: Construction of the barley construct.** A) The reporter gene mCHERRY (EC71091), an ER-targeting sequence (EC71090), a HDEL ER-targeting sequence (EC71020), and the 35S terminator (EC41414) were cloned into the Level 0 vector EC10161, using the Type IIS restriction enzyme *Bsa*I. The resulting Level 0.5 vector contains loxP sites flanking the inserted reporter gene and terminator. B) The maize Ubiquitin promoter *ZmUbi* with a 5’ UTR sequence (EC71139), *lox*P-flanked mCHERRY (EC10161), the ER-targeting sequence (EC71090), eGFP (EC71088), a HDEL ER-targeting sequence (EC71020), and the Actin2 terminator (EC41414) are cloned into a Level 1, position 3 vector (EC47822) in a reaction involving both *Bsa*I, for the canonical Level 0 and Level 1 parts, and *Esp*3I, for the *lox*P-containing Level 0.5 vector (see A).C) The HvHSP17::Cre-U5-Cre construct (EC71173), and the pGFP-LOX construct (EC71167), along with the position 4 end linker (EC41766), are cloned into a Level 2 construct (EC15027) which contains the 35s::Hygromycin selection cassette in position 1 using the Type IIS restriction enzyme *Bpi*I.


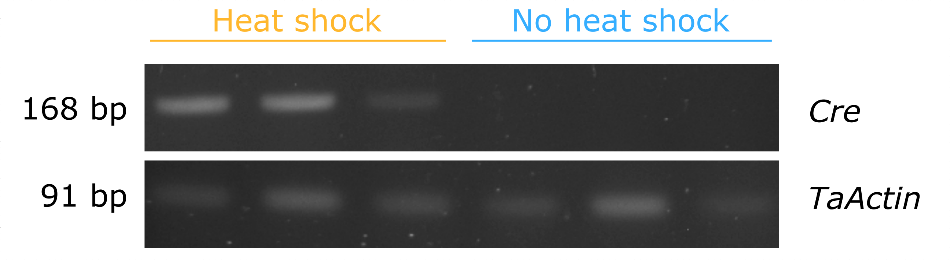


**Figure S4: *Cre* expression in HS_NAM-B1 transgenic lines.** RT-PCR was carried out on cDNA extracted from wheat seedlings which had undergone a two-hour heat shock treatment. Primers SH033 and SH002 were used to amplify *Cre* and primers PB47 and PB48 to amplify the housekeeping gene *TaActin*; see primer sequences in Supplementary Table 3. The expected band size was 168 bp for *Cre* and 91 bp for *TaActin*.


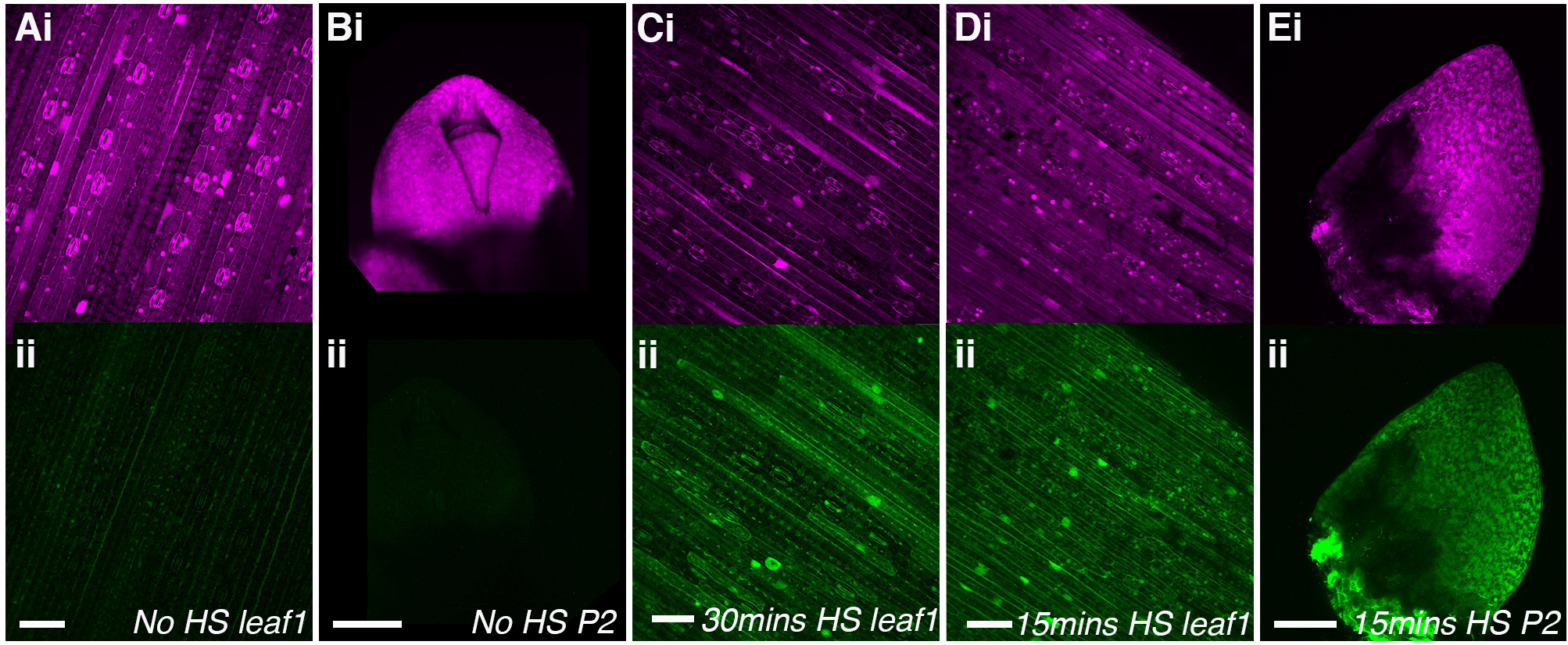


**Figure S5: Heat shock treatment of 00899-01-01.** 1-week-old seedlings grown on plates from the 00899-01-01 line were exposed to no heat shock (A-B), 30 minutes (C), or 15 minutes heat shock (D-E), and imaged 3 days later. Each panel shows the mCHERRY (magenta, i) and the eGFP (green, ii) expression. n=4. Scale bar: 100 μm.


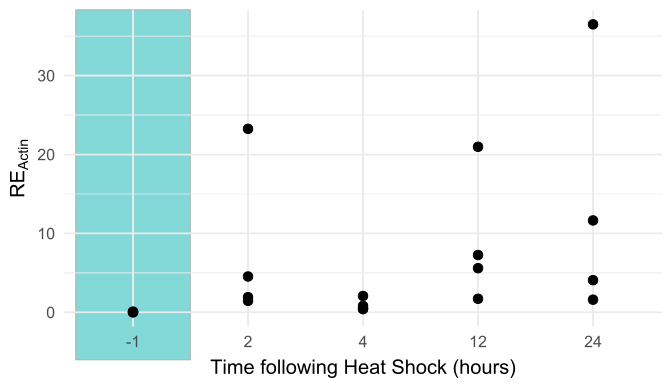


**Figure S6: Gene induction occurs within 2 hours following the termination of heat shock.** Wheat seedlings treated with a two-hour heat shock at 38 °C show significantly higher expression (p < 0.05) of *NAM-B1* than in samples taken one hour before heat shock (-1; blue). This is observed for all timepoints following heat shock (2, 4, 12, and 24 hours). There was no significant change in *NAM-B1* expression levels beyond two hours after heat shock (p > 0.05). The plants tested were from the single copy 2020-20-02 line. The expression levels of *NAM-B1* were determined using qRT-PCR and reported relative to the housekeeping gene *TaActin*. Statistical comparisons were carried out using the Wilcox test, with N = 4 for all conditions.

**
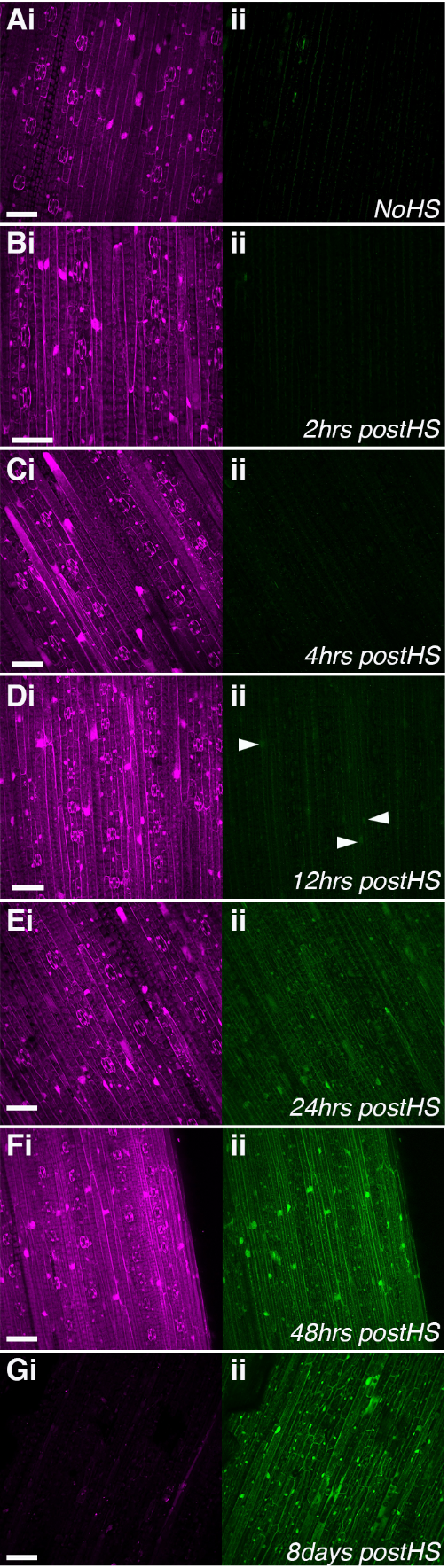
**

**Figure S7. Gene expression post heat shock treatment.** (A-F) GFP expression in leaf 1 of 1-week-old barley seedlings grown on plates with either, no heat shock (A) or 30minutes heat shock at 38°C (B-F). Each panel shows the mCHERRY (magenta, i) and the eGFP (green, ii) expression. Arrowheads highlight faint eGFP signal after 12 hours. Scale bar is 100 μm; n=4.


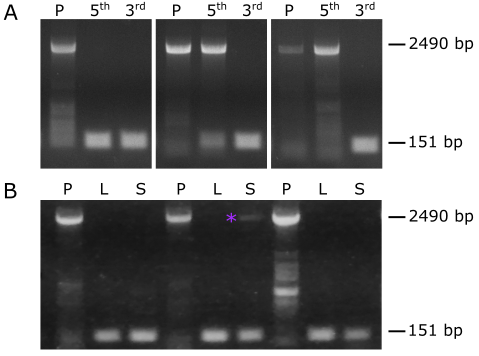


**Figure S8. Heat shock treatment does not always lead to complete excision of the reporter gene**. At the three-leaf stage (A), two hours of heat shock completely excises the reporter gene from the leaf tissue present at the time of heat shock (third leaf, “3^rd^”; 151 bp). Leaf tissue grown after completion of the heat shock treatment (fifth leaf, “5^th^”) is not always completely excised, as shown by the presence of the reporter gene (2490 bp band). Of fifteen individual plants tested using this assay, 6/15 showed full excision (left panel), 4/15 are intermediate (middle panel) and 5/15 have no excision (right panel). The three panels represent three independent biological replicates. (B) At flag leaf emergence, a two-hour heat shock at 38 °C leads to complete excision in the flag leaf (L) and in the corresponding basal spikelet (S) in most cases, though in some only partial excision is observed in the basal spikelet (purple asterisk). Of the 12 individuals tested, two showed partial excision. The reporter gene is present in all individuals before heat shock, with no evidence of premature excision (“P”). Gels shown are representative examples from (A) 15 T_1_ individuals derived from lines 2020-20-02, 2020-2-1, and 2020-19-01 (N = 5 for each line) and (B) 12 T_1_ individuals derived from lines 2020-2-1 and 2020-19-01 (N = 6 for each line).

**Table S1: Copy number of T_0_ transformants.** Lines taken forward for expression analysis and microscopy are highlighted in blue.

| **Construct** | **T_0_ Plant** | **Copies of Hygromycin** |
| --- | --- | --- |
| HS_NAM-B1 | 2020-20-01 | 46 |
|  | 2020-4-1 | 12 |
|  | 2020-19-01 | 10 |
|  | 2020-5-1 | 10 |
|  | 2020-13-1 | 4 |
|  | 2020-2-1 | 4 |
|  | 2020-8-1 | 4 |
|  | 2020-22-01 | 2 |
|  | 2020-23-01 | 2 |
|  | 2020-3-1 | 2 |
|  | 2020-21-01 | 1 |
|  | 2020-20-02 | 1 |
|  | 2020-24-01 | 1 |
|  | 2020-11-1 | 1 |
| HS_GFP | 00899-03-01 | 3-4 |
|  | 00899-02-01 | 2 |
|  | 00899-01-01 | 1 |
|  | 00899-04-01 | 1 |

**Table S2: Constructs used in this study.** All constructs are listed with their name as used in the text, and in relevant databases, as well as a short description. The level of the construct, corresponding to those described in Weber et al. 2011 (Levels 0-2) and Werner et al. 2012 (Level M), is also provided.

| **Name** | **Description** | **Origin** | **Construct** | **Level** |
| --- | --- | --- | --- | --- |
| pICH41421 | NosT | The Sainsbury Lab (TSL) Synbio^1^ | Wheat | 0 |
| pICSL12009 | ZmUbi promoter + ubiquitin 5’ UTR (untranslated 1^st^ exon + intron) | TSL Synbio^1^ | Wheat | 0 |
| pICH75111 | GUS (β-glucuronidase gene) with 2 introns | TSL Synbio^1^ | Wheat | 0 |
| pNAM-B1 | Domesticated NAM-B1, with 2 introns | This study | Wheat | 0 |
| pZmUbi-5'UTR | ZmUbi promoter + ubiquitin 5’ UTR (untranslated 1st exon + intron), with L0.5-compatible overhangs | This study | Wheat | 0 |
| pICH47751 | Level 1 Position 3 | TSL Synbio^1^ | Wheat | 1 |
| pICH47802 | OsAct promoter + Hygromycin coding sequence, L1P1 | TSL Synbio^1^ and BRACT^2^ (Rey et al. 2018) | Wheat | 1 |
| pNB1-LOX | ZmUbi::loxP-GUS-nosT-loxP-NAM-B1-nosT; L1P3 | This study | Wheat | 1 |
| pAGM8031 | Level M Position 1 | TSL Synbio^1^ | Wheat | M |
| pICH50892 | Level M, end linker 3 | TSL Synbio^1^ | Wheat | MEL |
| pLOXp-GUS | loxP-GUS-nosT-loxP | This study | Wheat | 0.5 |
| HS_NAM-B1 | Level M, P1. OsAct::Hyg-nosT/HvHSP17::Cre-hspT/ZmUbi::loxP-GUS-loxP-NAM-B1-nosT |  |  |  |
| EC10161 | LoxP Vector | This study | Wheat and Barley | 0 |
| EC71173 | Level 1 P2 HspHv17::Cre-U5-Cre | This study | Wheat and Barley | 1 |
| EC47822 | L1 vector backbone pL1V-R3-47822 | ENSA^3^ | Barley | 1 |
| EC71139 | P-pZmUBI-intron L0 | This study | Barley | 0 |
| EC71022 | U-LoxP-mCHERRY-HDEL-t35S-loxP. L0.5 | This study | Barley | 0.5 |
| EC71090 | pL0M-S-ER-Targ_71090 | ENSA^3^ | Barley | 0 |
| EC71088 | pL0M-C1-eGFP-71088 | ENSA^3^ | Barley | 0 |
| EC71020 | pL0M-C2-HDEL-71020 HDEL | ENSA^3^ | Barley | 0 |
| EC44300 | T-Act2 | ENSA^3^ | Barley | 0 |
| EC41414 | T-35S | ENSA^3^ | Barley | 0 |
| EC71091 | pL0M-C1-mCherry | ENSA^3^ | Barley | 0 |
| EC71102 | Unmodified CRE L0. pL0M-SC-Cre | This study | Barley | 0 |
| EC71167 | pL1M-R3-UbqP-Loxp-ER-Targ-mCherry-HDEL-Loxp-eGFP | This study | Barley | 1 |
| EC71174 | pL2M-HvHSP17-CREU5-UBQ-loxmCHERRYER-eGFPER | This study | Barley | 2 |
| EC47811 | L1 vector backbone pL1V-R2-47811 | ENSA^3^ | Barley | 1 |
| EC71100 | pL0M-PU-pHvHSP17 | This study | Barley | 0 |
| EC71171 | CRE with U5 intron at 254bp, in pICH41308 backbone | This study | Barley | 0 |
| EC15320 | T-AtHsp | ENSA^3^ | Barley | 0 |
| pICSL80006 | Turbo GFP with intron from the Arabidopsis U5 small nuclear ribonucleoprotein component gene | TSL Synbio^1^ | Barley | 0 |
| pICH41308 | Vector backbone for new CRE with intron | TSL Synbio^1^ | Barley | 0 |
| EC15027 | Level 2 Vector, including Hygromycin cassette at position 1 | ENSA^3^ | Barley | 2 |
| EC41766 | Level 2, end linker 3, pL1M-ele-3-41766 | ENSA^3^ | Barley | 2EL |

^1^https://www.synbio.tsl.ac.uk. Note that access to the database must be requested from the platform. Alternatively, most plasmids are available to be ordered from Addgene.

^2^<https://www.jic.ac.uk/research-impact/technology-platforms/genomic-services/crop-transformation/>

^3^https://www.ensa.ac.uk/resources (Note that ENSA has no facility for directly distributing constructs, however ENSA will put interested researchers in contact with those who can distribute the constructs.)

**Table S3: Primers used in the course of this study.** Calculated primer efficiencies for qRT-PCR are given where appropriate. For Level 0 cloning primers, the enzyme recognition sites are highlighted in blue, the resulting 4 base-pair overhangs are highlighted in red, and, where applicable, the Level 1 fusion sites are highlighted in bold.

| **Primer** | **Description** | **Sequence (5′ - 3′)** | **Reference** | **Efficiency** |
| --- | --- | --- | --- | --- |
| *gDNA_F* | Construct Excision PCR | CGATGCTCACCCTGTTGTTT | This study | NA |
| *gDNA_R* | Construct Excision PCR | GGTGATACCGCGTTGCTTTT | Borrill, P. (2013) | NA |
| *TaActin_F* | Actin, forward | ACCTTCAGTTGCCCAGCAAT | Uauy et al. (2006b) | 102% |
| *TaActin_R* | Actin, reverse | CAGAGTCGAGCACAATACCAGTTG | Uauy et al. (2006b) |  |
| *NAM-B1_F* | Domesticated NAM-B1, forward | AGTTGAACGGGGTCGACGAT | This study | 89% |
| *NAM-B1_R* | Domesticated NAM-B1, reverse | CTGCTGCCTCTCTCAGGTTG | This study |  |
| *Cre_F* | Cre recombinase, forward | ACCGGCATCAACGTTTTCTT | This study | NA |
| *Cre_R* | Cre recombinase, reverse | AAATGCTCCTGTCCGTTTGC | This study | NA |
| NAMB1_Level0_Forward_AATG | Level 0 cloning of NAM-B1 | ACGAAGACATCTCA**AATG**GGCAGCTCCGACTCATC | This study | NA |
| NAMB1_Level0_Reverse_GCTT | Level 0 cloning of NAM-B1 | ACGAAGACATCTCG**AAGC**TCAGGGATTCCAGTTCACGC | This study | NA |
| ZmUbi_Level0_Forward_GGAG | Level 0 cloning of ZmUbi | ATGAAGACATCTCA**GGAG**GTGCAGCGTGACCCGGTCGT | This study | NA |
| ZmUbi_Level0_Reverse_TACT | Level 0 cloning of ZmUbi | ACGAAGACATCTCG**AGTA**CCTGCAGAAGTAACACCAAA | This study | NA |
| Cre_E1_Level0_Forward_AATG | Level 0 cloning of Cre-U5-Cre | TGAAGACCAAATGTCCAATTTACTGACCGTACAC | This study | NA |
| Cre_E1_Level0_Reverse_AGGT | Level 0 cloning of Cre-U5-Cre | CGAAGACTTACCTGAAGATATAGAAGATAATCGC | This study | NA |
| Cre_I1_Level0_Forward_AGGT | Level 0 cloning of Cre-U5-Cre | CGAAGACTCAGGTAAGTTTCTGCTTCTACCTTTGATA | This study | NA |
| Cre_I1_Level0_Reverse_GCAG | Level 0 cloning of Cre-U5-Cre | AGAAGACGCCTGCACATCAACAAATTTTGGTCAT | This study | NA |
| Cre_E2_Level0_Forward_GCAG | Level 0 cloning of Cre-U5-Cre | AGAAGACGTGCAGGCGCGCGGTCTGGCAGTAA | This study | NA |
| Cre_E2_Level0_Reverse_GCTT | Level 0 cloning of Cre-U5-Cre | GGAAGACCAAAGCCTAATCGCCATCTTCCAGCAG | This study | NA |
